# Integrating LiDAR Sensor Data into Microsimulation Model Calibration for Proactive Safety Analysis

**DOI:** 10.3390/s24134393

**Published:** 2024-07-06

**Authors:** Morris Igene, Qiyang Luo, Keshav Jimee, Mohammad Soltanirad, Tamer Bataineh, Hongchao Liu

**Affiliations:** Department of Civil, Environmental and Construction Engineering, Texas Tech University, Lubbock, TX 79409, USA; morris.igene@ttu.edu (M.I.); qiyang.luo@ttu.edu (Q.L.); kjimee@ttu.edu (K.J.); mohammad.soltanirad@ttu.edu (M.S.); tamer.bataineh@ttu.edu (T.B.)

**Keywords:** LiDAR sensor, microsimulation, calibration, SSAM, proactive safety

## Abstract

Studies have shown that vehicle trajectory data are effective for calibrating microsimulation models. Light Detection and Ranging (LiDAR) technology offers high-resolution 3D data, allowing for detailed mapping of the surrounding environment, including road geometry, roadside infrastructures, and moving objects such as vehicles, cyclists, and pedestrians. Unlike other traditional methods of trajectory data collection, LiDAR’s high-speed data processing, fine angular resolution, high measurement accuracy, and high performance in adverse weather and low-light conditions make it well suited for applications requiring real-time response, such as autonomous vehicles. This research presents a comprehensive framework for integrating LiDAR sensor data into simulation models and their accurate calibration strategies for proactive safety analysis. Vehicle trajectory data were extracted from LiDAR point clouds collected at six urban signalized intersections in Lubbock, Texas, in the USA. Each study intersection was modeled with PTV VISSIM and calibrated to replicate the observed field scenarios. The Directed Brute Force method was used to calibrate two car-following and two lane-change parameters of the Wiedemann 1999 model in VISSIM, resulting in an average accuracy of 92.7%. Rear-end conflicts extracted from the calibrated models combined with a ten-year historical crash dataset were fitted into a Negative Binomial (NB) model to estimate the model’s parameters. In all the six intersections, rear-end conflict count is a statistically significant predictor (*p*-value < 0.05) of observed rear-end crash frequency. The outcome of this study provides a framework for the combined use of LiDAR-based vehicle trajectory data, microsimulation, and surrogate safety assessment tools to transportation professionals. This integration allows for more accurate and proactive safety evaluations, which are essential for designing safer transportation systems, effective traffic control strategies, and predicting future congestion problems.

## 1. Introduction

Microsimulation has gained widespread adoption due to its risk-free nature and cost-effectiveness. It is a powerful tool for modeling traffic operations and conducting safety studies. Simulation models replicate individual drivers’ behaviors within a virtual environment by providing a detailed representation of car-following and lane-changing behaviors in response to the surrounding traffic [1]. Compared to analytical models, microsimulation models provide individual vehicle movements on a second or sub-second basis [2]. A recent study on path dispersion (the spatial distribution of vehicular paths) [3] introduced a simulation model that describes the paths of left-turning vehicles under the unprotected left-turn phase inside intersections. The model analyzes drivers’ behavior to determine vehicle paths endogenously, rather than relying on predefined inputs. Due to the complexity of the path dispersion (which may be affected by geometric conditions, signal control, traffic conditions, and driver characteristics), the proposed microscopic traffic flow model is a starting point for future studies. Existing commercial microsimulation models including VISSIM, AIMSUN, CORSIM, and SUMO incorporate independent parameters with default values to describe traffic flow characteristics. These default values often yield unreliable results because driver behavior varies significantly depending on the geographical region and driving conditions. Thus, users frequently fine-tune these parameters to accurately represent traffic conditions specific to their case studies. Consequently, calibrating and validating microscopic simulation models is crucial for analyzing transportation alternatives, designing traffic control strategies, predicting future congestion problems, evaluating advanced vehicle technology impacts, performing safety analysis, and so on. Model calibration aims to minimize discrepancies between field measurements and simulation results. The accuracy of microsimulation models heavily relies on how well they are calibrated using available real-world traffic data [4].

Studies have shown that vehicle trajectory data are effective for calibrating microsimulation models [5]. Vehicle data can be collected from several sources, such as loop detectors [6], microwave radars [7], video cameras [1], or roadside LiDAR infrastructure [8,9,10]. Each one has its major strengths and weakness. Loop detectors are embedded in the road surface and count vehicles, measure speed, and detect occupancy. They are reliable for traffic volume data but provide limited spatial resolution and cannot capture detailed vehicle trajectories. Microwave radar sensors can detect vehicle speed and count and can operate in various weather conditions. However, they also provide limited trajectory data compared to more advanced methods. Video cameras are commonly used for obtaining detailed vehicle trajectory data and can capture a wide field of view. They have high resolution and can cover wide areas. However, video cameras have several weaknesses including high computing and processing requirements for real-time applications, sensitivity to weather conditions, and video quality is significantly affected by illumination condition [11]. LiDAR (Light Detection and Ranging) offers high-resolution 3D data, allowing for detailed mapping of the surrounding environment, including road geometry, roadside infrastructures, and moving objects such as vehicles, cyclists, and pedestrians. Unlike video cameras which capture detailed images that can reveal Personally Identifiable Information (PII) such as facial features or license plates, LiDAR represents objects by point clouds that consist of spatial data points. LiDAR’s high-speed data processing, fine angular resolution, and high measurement accuracy makes it well suited for applications requiring real-time response, such as autonomous vehicles [12]. They also perform well in bad weather and low-light conditions [13]. Additionally, with the continued development of LiDAR technology and its widespread use, the cost has significantly decreased over the past few years [14]. A complete simulation model has complex data requirements and numerous model parameters. There are significant challenges in effectively calibrating and validating these integrated models to ensure their reliability and accuracy in predicting safety outcomes. But past studies suggest that the reliability of microscopic traffic simulations can be substantially improved through rigorous calibration techniques [15].

This research aims to contribute to the ongoing efforts to improve transportation safety by presenting a comprehensive framework for integrating LiDAR sensor data into carefully calibrated simulation models for proactive safety analysis. The unique contributions of this study to the existing literature are multifaceted: (i) While LiDAR technology has been extensively used in autonomous vehicle development, its integration into traffic microsimulation models for safety analysis is relatively new. This study leverages high-resolution 3D data from LiDAR to extract precise vehicle trajectory information, which enhances the accuracy of simulation models. (ii) The study presents a rigorous calibration framework using the Directed Brute Force method to fine-tune car-following and lane-changing parameters in the Wiedemann 1999 model within VISSIM. This approach addresses the variability in driver behavior across different geographical regions and driving conditions, resulting in more reliable simulation outcomes. (iii) By combining LiDAR-derived vehicle trajectory data with historical crash data, this research advances the use of microsimulation for proactive safety analysis. The calibrated models identify rear-end conflicts and predict crash frequencies using a Negative Binomial model, providing a robust method for safety evaluation. Overall, by leveraging the strengths of LiDAR sensor data, this study sets a new standard for the accuracy and realism of microsimulation models, paving the way for more effective transportation safety evaluations and interventions. The rest of this paper is organized as follows: The Materials and Methods section provides details on the workflow for extracting vehicle trajectory data from LiDAR sensor point clouds, VISSIM model creation, the sensitivity analysis method, the calibration approach, and surrogate safety measures. The Results section presents results of calibration, the surrogate safety measures, and the Negative Binomial model. The Discussion section explains the results and their implications. Finally, the Conclusions section summarizes this paper.

## 2. Materials and Methods

**Figure 1:**
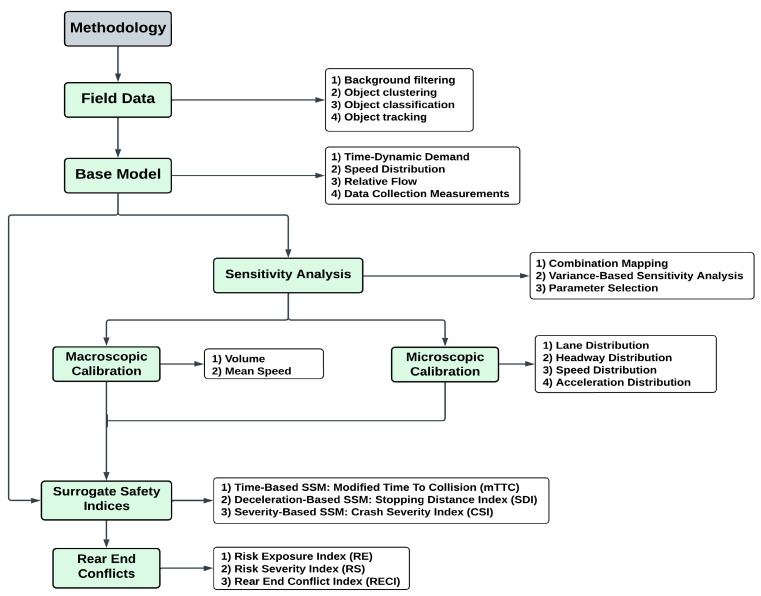
Workflow of the methodology.

This study implements a framework for integrating vehicle trajectory data obtained from LiDAR sensors into VISSIM models, performs a sensitivity analysis-based calibration, and predicts rear-end crashes obtained from surrogate safety assessment models. Three separate simulation models were developed, including the Default Model, Macro Model, and Micro Model. For the Default Model, no calibration was performed. Sensitivity analysis (SA) was performed to determine the significant parameters necessary for macroscopic and microscopic calibrations. Based on results of SA, two car-following and two lane-changing parameters of the Macro Models were calibrated using traffic volume and mean speed as Measures of Effectiveness (MOEs). Similarly, parameters of the Micro Models were calibrated using the distributions of lane, headway, speed, and acceleration as MOEs. Time-based (MTTC), deceleration-based (SDI), and severity-based (CSI) surrogate safety indices were calculated to estimate the Rear-End Conflict Index. Figure 1 illustrates the overall workflow of the methodology. Subsequent sections offer detailed explanations of these procedures.

### 2.1. LiDAR Sensor

**Figure 2:**
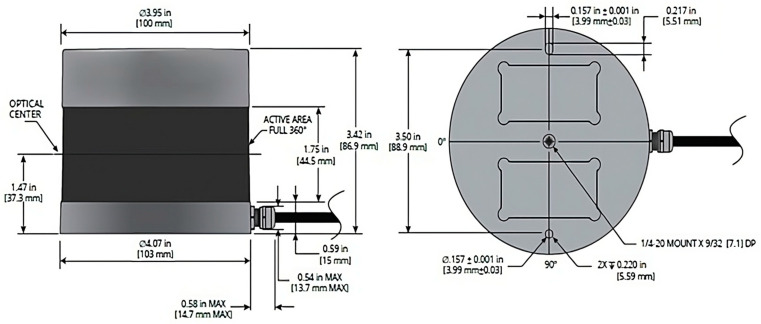
Parts and dimensions of a Velodyne VLP-32C LiDAR sensor.

LiDAR sensors capture data by emitting laser pulses and measuring the time it takes for the reflected light to return. This allows them to create a 3D representation of their surroundings as a point cloud. This 3D representation includes accurate information about objects’ shapes, sizes, and positions [16]. In this study, vehicle trajectory data were collected using a VLP-32 Velodyne LiDAR sensor (San Jose, CA). The VLP-32 LiDAR sensor is a 32-channel sensor. This means tha it emits 32 laser beams from a compact, spinning housing to create a 3D point cloud of the surrounding environment. With a range of 100 m (328 feet) and a rotational frequency set to 10 Hz, it captures 10 frames per second (records data every 0.1 s), each containing 600,000 3D points. The sensor has a full 360-degree horizontal field of view and a vertical field of view of 30 degrees (ranging from 15 degrees upwards to 15 degrees downwards). The VLP-32C LiDAR sensor components and their physical measurements are shown in Figure 2. Additionally, Table 1 lists several other specifications and characteristics of the sensor.

### 2.2. Field Data Collection

**Figure 3:**
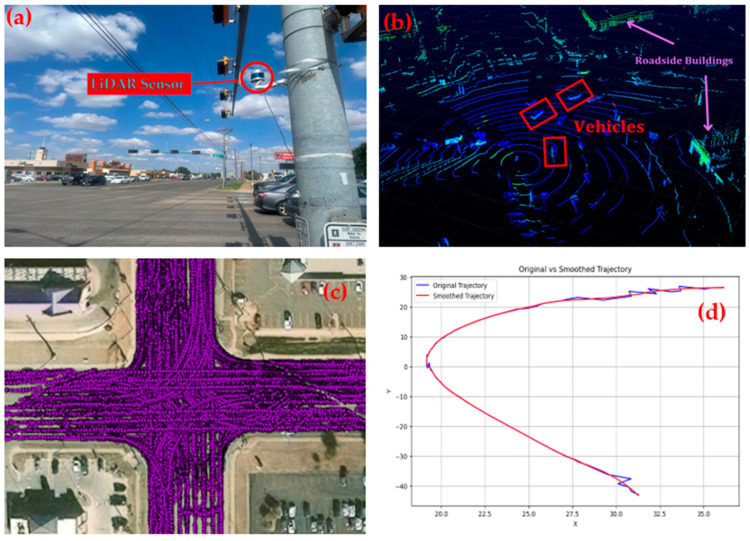
(**a**) A VLP-32 LiDAR sensor mounted on a traffic signal pole. (**b**) Raw 3D LiDAR sensor data visualized using Veloview. (**c**) Vehicle trajectories extracted from LiDAR sensor data. (**d**) A smoothed trajectory after removing noise.

Six urban signalized intersections in Lubbock, Texas, in the USA were selected in this study. These intersections include the following: (i) 34th Street and Indiana Avenue; (ii) 82nd Street and Milwaukee Avenue; (iii) 82nd Street and Slide Road; (iv) 50th Street and Avenue Q; (v) 50th Street and Quaker Avenue; and (vi) 4th Street and Frankford Avenue. The LiDAR sensor was mounted on a traffic signal pole at the corner of each intersection to record data. One of the study intersections is shown in Figure 3a, with the VLP-32 LiDAR sensor mounted on a pole. Data collection for each intersection lasted 2 h and was conducted during the evening peak commute period (4:00 pm to 6:00 pm) on different weekdays in March 2024.

### 2.3. Data Processing, Trajectory Extraction, and Smoothing

VeloView is a user application which provides real-time visualization of 3D LiDAR data from Velodyne LiDAR sensors. It is used to acquire, visualize, save, and replay sensor data. VeloView can also playback pre-recorded data stored in “pcap” (Packet Capture) files. Figure 3b shows the raw 3D point clouds obtained from the LiDAR sensor, visualized on the Veloview graphical user interface. 

A generalized four-step algorithm was developed by Zhao et al. (2019) for processing roadside-LiDAR sensor data [17]. The four steps include the following: (i) Background Filtering; (ii) Object Clustering; (iii) Object Classification; and (iv) Object Tracking. The first step employs a filtering technique based on point density to remove both static and dynamic background points from all frames. Step two involves clustering the remaining points into separate unique objects by means of a modified DBSCAN algorithm with varying parameters depending on their proximity to the LiDAR sensor. In step three, an artificial neural network (ANN) model is developed to classify the clustered objects into their respective road user groups, including vehicles, cyclists, and pedestrians. The parameters of the ANN model include distance to the LiDAR sensor, number of points in the cluster, and direction of cluster points distribution. Lastly, the Global Nearest Neighbor (GNN) is applied to track the same object across multiple data frames. The method proposed by Zhao et al. (2019) [17] was adopted in this study. Figure 3c shows vehicle trajectories extracted from the LiDAR sensor. A vehicle trajectory obtained from the sequence of processing algorithms is raw and frequently contains noise, deviations, and inconsistencies at the microscopic level (0.1 s), which can greatly affect the quality of the trajectory data. Therefore, it is necessary to smooth trajectory data to reduce the noise in the data (x and y-error correction). A recent study [18] introduces a new method for reconstructing 2D paths followed by objects, specifically driver behavior. However, the reconstructed trajectory using this method may be heavily impacted by outliers. In this study, the low pass Savitzky–Golay filter was used to denoise the trajectory data and accurately represent the vehicle’s intended path of travel. A window length of 11 and polyorder of 2 were used to smooth the raw trajectory data. The smooth trajectory data also removes the false peak in the velocity and acceleration profile, thereby providing the accurate representation of the velocity and acceleration profile. A detailed explanation of the trajectory data smoothing procedure can be found in the previous works of the authors [19]. Figure 3d shows the result of a smoothed trajectory. A sample of the processed trajectory data is presented in Table 1, showing all the variables used in this study.

### 2.4. Vissim Simulation Models

PTV’s **V**erkehr **I**n **S**tädten—**SIM**ulationsmodell (VISSIM 2024) was used to develop the simulation models for this study. VISSIM is based on a psycho-physical car-following model that incorporates perception thresholds to model driver behavior. The basic concept of the car-following model is that drivers are sensitive to the changes in distance and speed of slower moving vehicles in front of them [20]. PTV VISSIM is by far the most used traffic microsimulation software for simulation-based surrogate safety studies [15]. Researchers cited its high flexibility in simulating real-world traffic conditions and the ease of extracting conflict data without resorting to manual observation. Each of the six study intersections were modeled in VISSIM. The geometry includes lanes, link, design speed, designated turn lanes, vehicle storage lengths, and curb turn radii. These data were obtained from Geographic Information System (GIS) files and Google Maps. The processed vehicle trajectory data were used as vehicle input (volume) and static routing decision (relative flow). Signal timing sheets were obtained from the City of Lubbock for all six intersections. The PM peak day plan was used to code the signal control operation in the VISSIM network model of each study intersection [2]. Three separate simulation models were developed for each study intersection, including the Default Model, Macro Model, and Micro Model. For the Default Model, no calibration was performed (all the default parameters of the Wiedemann 99 model were retained). And the model was simulated for 2 simulation hours to replicate the duration of field data collection.

### 2.5. Sensitivity Analysis

Sensitivity analysis (SA) helps identify which model parameters have the most significant influence on the output. This information is crucial for calibration because it allows traffic engineers to focus their efforts on the most impactful parameters, making the calibration process more efficient and effective. Variance-based sensitivity analysis quantifies the importance of both individual factors (first-order effects) and interactions (total effects) in complex models. By analyzing both first-order effects and total effects, we gain a deeper understanding of how each factor contributes to the overall output variability. Consider a model with multiple factors affecting a single output, given in the form shown in Equation (1).

(1) $$Y=f\left(X_{1},X_{2},\ldots ,X_{d}\right)$$

First-order effects represent the influence of a single factor ($X_{1}$) on Y, independent of other factors. These are relatively easy to analyze using tools like regression. Interactions, on the other hand, capture the combined, non-additive effects of multiple factors and are more challenging to detect. Sobol, 1976 [21] initially introduced the variance decomposition method, offering both analytical derivation and Monte Carlo implementation. The core concept of Sobol decomposition lies in Sobol indices. These indices quantify the contribution of individual variables and their interactions to the variance of the output. There are two main types of Sobol indices: (1) the main effect (first order) index ($S_{i}$) which measures the individual contribution of a generic factor $X_{i}$ to the variance of Y, and (2) the total effect index ($S_{T}$) which captures main effect of $X_{i}$ plus its interaction effects with other variables. The latest advancements in Sobol 1976, useful for practical application, come from Saltelli et al., 2008 [22]. The expression of $S_{i}$ and $S_{T}$ are given in Equations (2) and (3).

(2) $$S_{i}=\frac{\frac{1}{N}\sum_{j=1}^{N}y_{B}^{\left(j\right)}\left(y_{C_{i}}^{\left(j\right)}-y_{A}^{\left(j\right)}\right)}{\left(\frac{1}{2N}\sum_{j=1}^{N}{\left(y_{A+B}^{\left(j\right)}\right)}^{2}-{\left(\frac{1}{2N}\sum_{j=1}^{N}y_{A+B}^{\left(j\right)}\right)}^{2}\right)}$$

(3) $$S_{T}=\frac{\frac{1}{2N}\sum_{j=1}^{N}{\left(y_{A}^{\left(j\right)}-y_{C_{i}}^{\left(j\right)}\right)}^{2}}{\left(\frac{1}{2N}\sum_{j=1}^{N}{\left(y_{A+B}^{\left(j\right)}\right)}^{2}-{\left(\frac{1}{2N}\sum_{j=1}^{N}y_{A+B}^{\left(j\right)}\right)}^{2}\right)}.$$

where A, B, and $C_{i}$ are matrices defined in Equations (4)–(6):

(4) $$A=\left[\begin{array}{cccc} X_{1}^{\left(1\right)} & X_{2}^{\left(1\right)} & \ldots & X_{k}^{\left(1\right)}\\ X_{1}^{\left(2\right)} & X_{2}^{\left(2\right)} & \ldots & X_{k}^{\left(2\right)}\\ \ldots & \ldots & \ldots & \vdots \\ X_{1}^{\left(N\right)} & X_{2}^{\left(N\right)} & \ldots & X_{k}^{\left(N\right)} \end{array}\right]$$

(5) $$B=\left[\begin{array}{cccc} X_{k+1}^{\left(1\right)} & X_{k+2}^{\left(1\right)} & \ldots & X_{2k}^{\left(1\right)}\\ X_{k+1}^{\left(2\right)} & X_{k+2}^{\left(2\right)} & \ldots & X_{2k}^{\left(2\right)}\\ \ldots & \ldots & \ldots & \vdots \\ X_{k+1}^{\left(N\right)} & X_{k+2}^{\left(N\right)} & \ldots & X_{2k}^{\left(N\right)} \end{array}\right]$$

(6) $$C_{i}=\left[\begin{array}{cccccc} X_{1}^{\left(1\right)} & X_{2}^{\left(1\right)} & \ldots & X_{k+i}^{\left(1\right)} & \ldots & X_{k}^{\left(1\right)}\\ X_{1}^{\left(2\right)} & X_{2}^{\left(2\right)} & \ldots & X_{k+i}^{\left(2\right)} & \ldots & X_{k}^{\left(2\right)}\\ \ldots & \ldots & \ldots & \ldots & \ldots & \vdots \\ X_{1}^{\left(N\right)} & X_{2}^{\left(N\right)} & \ldots & X_{k+i}^{\left(N\right)} & \ldots & X_{k}^{\left(N\right)} \end{array}\right]$$

For i=1,…,k.

A multistep approach for complex traffic simulation models’ sensitivity analysis (SA) was proposed by Ciuffo and Azevedo, 2014 [23]. In the first step, parameters are grouped with respect to the sub-models they are part of, and an SA is carried out considering the different groups rather than the different parameters. Then, the most influential groups on the model outputs are identified, and a new SA on all the parameters of these groups is carried out. 

In this study, a multistep SA was performed to identify car-following parameters of the Wiedemann 99 model (including lane-changing parameters) with significant effect on the output variance. Table 2 and Table 3 present all the parameters of the Wiedemann 99 chosen for sensitivity analysis. 

The first step of sensitivity analysis involves grouping parameters which measure the same quantity. SA is performed on each group separately to select the parameter with the most significant influence on the output variance. For instance, in the first step, CC0 and CC2 (m) are grouped because they both measure distance. Similarly, CC1 and CC3 (s) are combined because they both measure time. This iterative process is repeated for all parameter sets, measuring the same quantity. After a reduction in the parameters set, a final SA was performed to identify the subset of final model parameters which represent only the parameter with the most significant influence on the output variance. Based on SA results across the six study intersections, the following car-following and lane-change parameters were selected: Standstill Distance (CC0), Gap Time Distribution (CC1), Safety Distance Reduction Factor (SDRF), and Accepted Deceleration—Trailing (ADT). In a study by Buck et al. (2017), it was found that the CC0 parameter influences vehicles near the stop line at the front of the queue, whereas the CC1 parameter has an impact on vehicles located further back in the queue [24]. Before making a lane change, drivers consider the reduced safety distance resulting from such maneuver. In VISSIM, this reduction is calculated by multiplying the original safety distance by the Safety Distance Reduction Factor (SDRF). The resulting distance represents the minimum acceptable headway a vehicle needs in the neighboring lane to safely complete the lane change. Accepted Deceleration—Trailing (ADT) describes the specific deceleration rate that the vehicle in the new lane (the trailing vehicle) is willing to accept to allow the lane change to occur safely [25].

### 2.6. Calibration of Simulation Models

Researchers have treated the calibration of simulation models as optimization problems consisting of a searching algorithm and an objective function. The goal is to determine the optimal set of parameters that minimize the objective function. The Genetic Algorithm (GA) is among the most widely used metaheuristic methods by researchers in calibration/validation problems. This is due to its easy implementation and good performance in calibration and optimization. However, Hale et al. (2015) argued that GA frequently requires thousands of trials to locate an acceptable solution, and the traffic simulations cannot process thousands of trial runs in a reasonable time frame [26]. Recently, researchers have also implemented several other nonlinear optimization methods to solve calibration problems. Some of these methods include Simultaneous Perturbation Stochastic Approximation (SPSA) [27], sequential quadratic programming [28], dynamic time warping algorithm [29,30], artificial neural networks [31], cross-entropy [32], and Directed Brute Force (DBF) [26]. Yu and Fan, 2017 cited several goodness-of-fit formulas previously used by researchers to calibrate simulation models [4]. Hale et al. (2015) compared SPSA and the “directed brute force” (DBF) method search [26]. The results showed that although SPSA was faster than the DBF method, the results of DBF are more reliable. Multiple random seed number replications are executed for each combination of parameter values to obtain more statistically reliable output. The Macro Models were calibrated based on the method proposed by Hale et al., 2015 using traffic volume and mean speed as Measures of Effectiveness (MOEs) [26]. Similarly, the Micro Models were calibrated using the distributions of lane, headway, speed, and acceleration as MOEs. After calibration, each model was simulated for 2 simulation hours to replicate the duration of field data collection. 

### 2.7. Surrogate Safety Measures (SSMs)

Surrogate safety measures (SSMs) have been extensively used to detect near-crash events. SSMs measure the time and space proximity of a vehicle pair to the point of collision or detect their evasive actions to avoid a collision. SSMs serve as a proactive approach to road safety analysis without relying on historic crash data. Recently, there has been significant interest in SSMs due to the causal relationship between near-crash events identified by SSMs and the actual crashes from field observations. Traffic conflicts have been considered an effective surrogate safety measure. Compared to crashes, conflicts are much more frequent and easier to be captured in the real world [33].

The Surrogate Safety Assessment Model (SSAM) is an automated tool that has been widely used by researchers to extract surrogate measures such as Post Encroachment Time (PET), Time to Collision (TTC), and Modified Time to Collision (MTTC) from simulation models [34]. From the initial positions and velocities of the pair of vehicles, SSAM generates 100 paths for each vehicle using combinations of acceleration and orientation that are independently generated from two triangular distribution functions and detects all collision points between every pair of projected paths. In this study, SSAM-3.0 was used to extract rear-end conflict data from the VISSIM-simulated trajectory files.

Bhattarai et al., 2023 present a methodology based on three surrogate safety indices to detect rear-end conflicts at signalized intersections [16]. The authors combined time-based (MTTC), deceleration-based (SDI), and severity-based (CSI) surrogate safety indices to estimate the Rear-End Conflict Index (RECI) at the intersection of Los Altos Parkway and Pyramid Way in Reno, Nevada. The Stopping Distance Index (SDI) is a surrogate measure used to capture rear-end conflicts using Equations (7)–(9):

(7) $$SDI=\left\{\begin{array}{c} 1\;\left(unsafe\right)\;\forall {SD}_{l}\;\leq \;{SD}_{f}\\ 0\;\left(safe\right)\;\forall \;{SD}_{l}>{SD}_{f}\;\;\;\; \end{array}\right.$$

(8) $${SD}_{l}=\frac{\nu_{l}^{2}}{{2D}_{max}}+(\nu_{f}*P_{r})$$

(9) $${SD}_{f}=\frac{\nu_{l}^{2}}{{2D}_{max}}+h_{lf}$$

where

SDI = Stopping Distance Index.
${SD}_{l}$ = Stopping Distance of leading vehicle.
${SD}_{f}$ = Stopping Distance of following vehicle.
$\nu_{l}$ = Velocity of leading vehicle.
$\nu_{f}$ = Velocity of following vehicle.
$P_{r}$ = Perception-reaction time.
$D_{max}$ = Maximum Available Deceleration Rate.
$h_{lf}$ = Headway between the leading vehicle and the following vehicle.

AASHTO’s A Policy on Geometric Design of Highways and Streets [35] recommends a perception-reaction time ($P_{r}$) of 2.5 s. A Maximum Available Deceleration Rate ($D_{max}$) of ${8\;m/s}^{2}$ was recommended by a previous study [36].

The crash severity index (CSI) is quantified by the expression given in Equation (10) [37].

(10) $$CSI=\frac{1}{MTTC}*\frac{{\left(\nu_{f}+a_{f}*MTTC\right)}^{2}-{\left(\nu_{l}+a_{l}*MTTC\right)}^{2}}{2}$$

where

MTTC = Modified Time to Collision.
$a_{l}$ = Acceleration of leading vehicle.
$a_{f}$ = Acceleration of following vehicle.

MTTC values below a predefined threshold are combined with SDI = 1 (unsafe) to estimate the Risk Exposure (RE) index within each temporal window. Similarly, the MTTC values below the threshold are combined with CSI to estimate Risk Severity (RS) index values within the same temporal window. Equations (11) and (12) show their mathematical relationships.

(11) $$RE=\frac{U_{i}}{n_{i}}$$

where

RE = Risk Exposure.
$U_{i}$ = Unsafe instances (count of frames with MTTC < 2 s and SDI = 1).
$n_{i}$ = Total count of instances (frames).

(12) $$RS=\frac{{CSI}_{max}}{{CSI}_{critical}}$$

where

RS = Risk Severity.
${CSI}_{max}$ = maximum CSI value for the temporal window.
${CSI}_{critical}$ = maximum possible CSI value.

As each pair of vehicles interacts at an intersection, the RE and RS indices constantly assess their conflict risk at every temporal window. This combined effect is the Rear-End Conflict Index (RECI), obtained by normalizing the RE and RS indices with min-max scaling using Equations (13)–(15).

(13) $$RECI={RE}_{norm}*{RS}_{norm}$$

(14) $${RE}_{norm}=\frac{{RE}_{i}-min({RE}_{1},\ldots ,{RE}_{N})}{max({RE}_{1},\ldots ,{RE}_{N})-min({RE}_{1},\ldots ,{RE}_{N})}$$

(15) $${RS}_{norm}=\frac{{RS}_{i}-min({RS}_{1},\ldots ,{RS}_{N})}{max({RS}_{1},\ldots ,{RS}_{N})-min({RS}_{1},\ldots ,{RS}_{N})}$$

where

RECI = Rear-End Conflict Index.
${RE}_{norm}$ = Normalized RE.
${RS}_{norm}$ = Normalized RS.
i = Current window.
N = Total count of windows.

## 3. Results

### 3.1. Field Data

Field data were collected at each of the six study intersections in Lubbock, Texas, USA: (i) 34th Street and Indiana Avenue, (ii) 82nd Street and Milwaukee Avenue, (iii) 82nd Street and Slide Road, (iv) 50th Street and Avenue Q, (v) 50th Street and Quaker Avenue, and (vi) 4th Street and Frankford Avenue. The study period covers the evening peak commute period (4:00 pm to 6:00 pm) on six different weekdays in March 2024. 

### 3.2. Sensitivity Analysis and Model Calibration Results

The results of the sensitivity analysis (SA) include the main effect ($S_{i}$), which measures the individual contribution of each parameter to the output variance and the total effect ($S_{T}$), which captures the main effect plus its interaction with other parameters. ($S_{i}$) and ($S_{T}$) were calculated using Equations (2) and (3), respectively. Based on the results of the SA, the following car-following and lane-change parameters were selected: Standstill Distance (CC0), Gap Time Distribution (CC1), Safety Distance Reduction Factor (SDRF), and Accepted Deceleration—Trailing (ADT). Based on findings from the literature [1,38] and early experimentation in VISSIM, the coefficients considered for the Wiedemann 99 car-following model parameters and lane-changing parameters are given below.

- CC0: 1.6 ft to 6.7 ft @ 0.1 ft step.
- CC1: 0.7 s to 1.0 s @ 0.1 s step.
- SDRF: 0.1 to 0.7 @ 0.1 step.
- ADT: −6.56, −3.28 and −1.64 (ft/s^2^).

The model calibration was treated as optimization problems consisting of a search algorithm and an objective function. The objective function was to minimize the difference between field-observed measures and calibrated measures (e.g., field-observed speed vs. simulated speed). This can be achieved by identifying the optimum combination of the coefficients of the selected lane-changing and car-following parameters. For the Macro Models, Root Mean Squared Error (RMSE) was selected as the goodness-of-fit measure between simulated and observed performance measures. To ensure that the error metrics are scale-independent, the RMSE values were normalized values between 0 and 1 based on the minimum and maximum observed values of volume and mean speed. The objective function for the Micro Models is extended to consider not just volume and mean speed but also lane usage and traffic characteristics (distributions of vehicle headway, speed, and acceleration). A confusion matrix is used to compare how well the simulated lane usage matches the observed lane usage. The confusion matrix shows how many vehicles were assigned to each lane category in both simulated and observed data. Additionally, the Kolmogorov–Smirnov test was used to compare the distributions of vehicle headway, speed, and acceleration from both simulated and observed data and calculate *p*-values. The *p*-value indicates the probability that the distributions are statistically different. Overall, this modified objective function provides a more comprehensive evaluation of the traffic simulation by considering not just volume and speed but also lane usage patterns and the statistical distribution of traffic characteristics. This can lead to a more robust optimization process that calibrates the simulation to reflect real-world traffic behavior better. The collected dataset was split in the order of 1.5 h (frame 0–53,999) and 0.5 h (frame 54,000–71,999), representing the 75% calibration and 25% validation sets, respectively. After calibrating and validating the model, default coefficients of the calibrated parameters in VISSIM were then replaced with the identified coefficients to produce a simulation of the real-world scenario. The calibrated parameters for the Macro and Micro Models across all six study intersections are shown in Table 4 and Table 5, respectively. 

The experimental study evaluates the accuracy of traffic volume simulations at various intersections using three different models: Default Model, Macro Model, and Micro Model. Observed volumes (veh/h) are compared with simulated volumes, and the accuracy percentage is calculated for each model. Table 6 presents the results of the observed versus VISSIM-simulated traffic volumes.

- **Default Model**: The Default Model shows varying accuracy, with the highest accuracy of 99.7% at the 82nd Street and Slide Road intersection and the lowest accuracy of 91.6% at the 4th Street and Frankford Avenue intersection.
- **Macro Model**: The Macro Model consistently provides high accuracy, ranging from 99.3% to 99.9%, demonstrating its reliability across different intersections.
- **Micro Model**: The Micro Model also demonstrates high accuracy, with the lowest accuracy being 99.5% at the 50th Street and Quaker Avenue intersection and the highest accuracy of 99.9% at multiple intersections.

Similarly, the study evaluates the accuracy of mean speed simulations at various intersections using Default Model, Macro Model, and Micro Model. Observed mean speeds (mi/h) are compared with simulated speeds, and the accuracy percentage is calculated for each model. Results of observed mean speed and simulated mean speeds are presented in Table 7.

- **Default Model**: The Default Model shows moderate accuracy, with the highest accuracy of 88.5% at the 50th Street and Quaker Avenue intersection and the lowest accuracy of 69.2% at the 34th Street and Indiana Avenue intersection.
- Macro Model: The Macro Model has improved accuracy over the Default Model, ranging from 75.4% to 92.3%, indicating better performance across different intersections.
- Micro Model: The Micro Model demonstrates the highest accuracy among the models, with the lowest accuracy being 80.8% at the 50th Street and Avenue Q intersection and the highest accuracy of 97.4% at the 82nd Street Slide Road intersection.

The Default Model shows less consistent accuracy compared to the Macro and Micro Models, indicating room for improvement. The Micro Model consistently provides the highest accuracy of simulated volume and mean speed, making it the most reliable.

### 3.3. Conflict Extraction

The Surrogate Safety Assessment Model (SSAM) was used to directly extract simulated conflict data from trajectory (.trj) files generated from PTV VISSIM. SSAM follows a four-step procedure to extract conflicts from simulated trajectory data. (i) **Define the Analysis Area:** First, SSAM reads the header information in the TRJ file to determine the dimensions of the analysis area. It then constructs a zone grid that completely covers this rectangular area. (ii) **Project Vehicle Paths:** For each vehicle within the analysis region, SSAM projects its expected location based on its current speed. This projection assumes that the vehicle continues traveling along its future path for a set duration, typically 1.5 s, using trajectory data for the next 10 s to perform this projection. (iii) **Identify Potential Conflicts:** SSAM calculates a rectangular perimeter that defines the location and orientation of each vehicle at its projected future position. This rectangle is then overlaid on the zone grid. SSAM identifies which zones in the grid will contain at least part of the vehicle based on this overlap. For each zone containing the projected vehicle, SSAM checks for an overlap between the new vehicle’s rectangle and any other vehicles (rectangles) already present in that zone. (iv) **Refine Time-to-Collision (TTC):** For any identified overlapping vehicle pairs, SSAM refines the initial TTC value. It does this by iteratively shortening the future projection timeline by tenths of a second. For each shortened timeframe, both vehicles are re-projected as before for successively shorter distances. This iterative process allows for a more accurate calculation of the TTC for that specific time step. Finally, various surrogate safety measures are calculated and updated based on these refined TTC values [34]. Generally, SSAM conflicts are classified into only three types: crossing, rear-end, and lane change. This study focusses on rear-end crashes at signalized intersections. Therefore, rear-end conflicts were selected for further analysis. Rear-end conflict pairs extracted from each study intersection are presented in Table 8.

### 3.4. Surrogate Safety Measures

**Figure 4:**
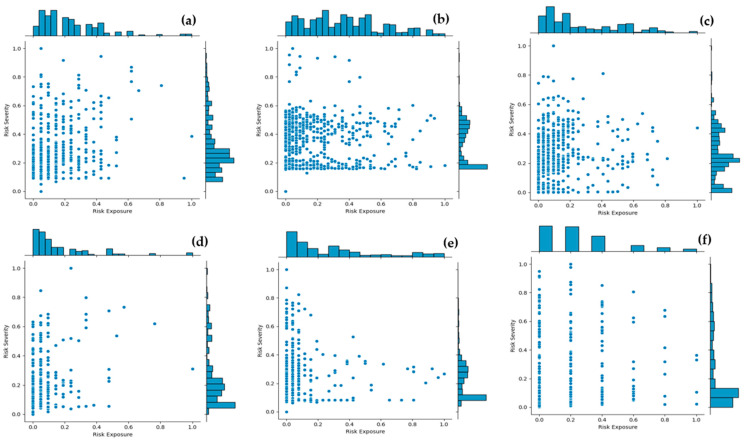
(**a**) 34th/Indiana; (**b**) 82nd/Milwaukee; (**c**) 82nd/Slide; (**d**) 50th/Avenue Q; (**e**) 50th/Quaker; (**f**) 4th/Frankford.

There are several factors that could potentially explain the differences in conflict counts across the study intersections. First, all six study intersections are four-leg intersections. Based on the signal timing sheets, all six intersections have the same cycle length of 130 s during the PM peak period. It is observed that 82nd Street/Milwaukee Avenue and 82nd Street/Slide Road have dual left turn lanes, and therefore run protected left turn phasing, while others have a single left turn lane each and run protected-permitted phasing during the PM peak commute period. Finally, the results of data collection show that traffic volume varies significantly across all six study intersections. The observed traffic volume can also explain the differences in conflict counts. For all rear-end conflict pairs extracted from SSAM, the corresponding MTTC value was used to calculate SDI and CSI. The Risk Exposure (RE) index and Risk Severity (RS) index were calculated using Equations (11) and (12) for every 1 s temporal window. Only the surrogate safety measures from Micro Models are provided in this paper because they performed much better than the other two models. This is consistent with previous studies [39]. Figure 4 shows the distribution of normalized RS and RE indices obtained using Equations (14) and (15), respectively.

Normalized RE and RS indices are shown in the central plot area, while frequency distributions of the observations are shown in the marginal plots. The frequency distribution of the normalized RE index shows heavy volume at the range between 0 and 0.6, with the tail capturing the higher exposure indices above 0.8 to 1. However, the distribution of the normalized RS index shows heavy volume at the lowest range between 0 and 0.45, with the tail capturing the higher severity indices above 0.5 to 1. The 15th percentile from the top of the distributions of normalized RE and normalized RS were used to evaluate the Rear-End Conflict Index (RECI) from Equation (13).

### 3.5. Rear-End Crash Prediction

Crash records of the past ten years (2014–2023) were collected from the Crash Records Information System (CRIS). CRIS is a crash database of the Texas Department of Transportation (TxDOT). The annual crash frequencies were extracted from all six intersections at a radius of 328 feet, matching the detection range of the LiDAR sensor. The crash data were filtered based on vehicle travel directions to extract the rear-end crashes. The result was further streamlined based on the hour of day to obtain only the crashes corresponding to the data collection period (4:00 PM to 6:00 PM). A negative binomial (NB) model was developed to estimate the expected number of crashes based on rear-end conflict at hourly time intervals. The NB model has been used in previous crash-prediction studies because it accounts for the over-dispersion in crash data resulting from unobserved heterogeneity [40]. Negative Binomial models were developed with rear-end conflicts identified at 95th percentile values of the RECI index. The ten years’ historic crash data and the rear-end conflicts were fitted into a Negative Binomial regression model to analyze the relationship between identified conflicts and crash frequency. Table 9 presents the results of Negative Binomial models across the six study intersections.

The rear-end conflict count coefficient represents the effect of rear-end conflicts on the outcome. Positive coefficients indicate an increase in rear-end conflicts resulting in an increase in crash frequency. Pseudo R-squared (Cox and Snell) measures the proportion of variation in the dependent variable explained by the model. Higher values indicate better model fit. *p*-value assesses the significance of the rear-end conflict count coefficient. *p*-values below 0.05 indicate statistically significant effects. A lower AIC indicates a better model fit, considering the number of predictors. Log-likelihood is a statistical measure used to assess how well a model fits the data. In this case, lower log-likelihood values indicate a better fit. While it is not directly comparable across models due to potential scaling differences, we can see that all intersections have negative log-likelihood values, suggesting a reasonable fit for the data. Deviance is another measure of model fit, related to log-likelihood. It represents the difference between the model and a perfect model (where the predicted values perfectly match the observed data). Lower deviance values indicate a better fit. Here, we see a similar trend to log-likelihood, with all intersections having relatively low deviance values.

For intersections 34th/Indiana, 82nd/Milwaukee, 50th/Ave Q, 50th/Quaker, and 4th/Frankford, the rear-end conflict count is a statistically significant predictor (*p*-value < 0.05). For 82nd/Slide, the *p*-value is 0.05, indicating marginal significance. The model explains a significant proportion of the variance in the dependent variable for all intersections, with pseudo R-squared values ranging from 0.2142 to 0.6515. The best model fit is for 34th/Indiana (pseudo R-squared = 0.6515). The impact of rear-end conflicts varies by location. The coefficient is highest for 50th/Quaker (1.5677), indicating a strong effect, and lowest for 82nd/Slide (0.0165). Based on AIC values, the model for 4th/Frankford has the best fit (AIC = 16.669), suggesting that it balances model complexity and goodness-of-fit most effectively.

## 4. Discussion

The results of the distribution of RE indices indicate that there are a lot of instances with high-risk exposure (up to 0.6) across the six study intersections. But the corresponding RS indices show heavy volume at the lowest range between 0 and 0.45. This explains the scenarios at intersection approaches where exposure is high due to vehicles decelerating or stopping in response to traffic signals, but the risk of such conflict is very low due to reduced or zero speed. Therefore, extreme values of RE and RS were selected to capture instances that represent dangerous driving behaviors. The top 15th percentile values of RE and RS were selected to estimate the Rear-End Conflict Index (RECI) at each 1 s temporal window. Negative Binomial models were developed with rear-end conflicts identified at 95th percentile values of the RECI. The ten years’ historic crash data and the rear-end conflicts were fitted into a Negative Binomial regression model to analyze the relationship between identified conflicts and crash frequency.

### 4.1. 34th Street and Indiana Avenue

The positive coefficient for rear-end conflict count (0.2214) suggests that an increase in rear-end conflict count is associated with an increase in the expected Crash Count. Specifically, for each unit increase in the rear-end conflict count, the log of the expected Crash Count increases by 0.2214. The intercept value of −2.2513 indicates the log of the expected Crash Count when the rear-end conflict count is zero. The model’s pseudo R-squared value of 0.6515 indicates that the model explains approximately 65.15% of the variance in the Crash Count. The rear-end conflict count is a highly significant predictor of the Crash Count (*p*-value < 0.001). The AIC value of 28.4242 provides a measure of the model’s goodness of fit.

### 4.2. 82nd Street and Milwaukee Avenue

The coefficient for the rear-end conflict count (0.0423) is positive. For each unit increase in the rear-end conflict count, the log of the expected Crash Count increases by 0.0423. The intercept value is −2.2524. The model’s pseudo R-squared value of 0.5496 indicates that the model explains approximately 54.96% of the variance in the Crash Count. The rear-end conflict count is a highly significant predictor of the Crash Count (*p*-value < 0.001).

### 4.3. 82nd Street and Slide Road

The rear-end conflict count (0.0165) suggests a positive relationship with the Crash Count, but its statistical significance is marginal (*p*-value = 0.053). The intercept value is −1.0561. The model’s pseudo R-squared value of 0.2142 indicates a relatively weak fit compared to the previous models, suggesting that the model explains only about 21.42% of the variance in Crash Count. While the rear-end conflict count shows a positive relationship with the Crash Count, its significance level is borderline (*p*-value = 0.053).

### 4.4. 50th Street and Avenue Q

The coefficient for the rear-end conflict count is 0.6979. The intercept value is −1.7797. The model’s pseudo R-squared value of 0.3768 suggests that the model explains approximately 37.68% of the variance in the Crash Count. The rear-end conflict count is a highly significant predictor of the Crash Count (*p*-value < 0.001).

### 4.5. 50th Street and Quaker Avenue

The rear-end conflict count (1.5677) suggests that an increase in the rear-end conflict count is associated with a substantial increase in the expected Crash Count. The intercept value is −0.8329. The model’s pseudo R-squared value of 0.3540 suggests that the model explains approximately 35.40% of the variance in the Crash Count. The conflict count is a statistically significant predictor of the Crash Count (*p*-value = 0.018).

### 4.6. 4th Street and Frankford Avenue

The coefficient of the rear-end conflict count is 0.4993 with an intercept value of −4.7538. The model’s pseudo R-squared value of 0.5834 indicates a strong fit, suggesting that the model explains approximately 58.34% of the variance in the Crash Count. The rear-end conflict count is a statistically significant predictor of the Crash Count (*p*-value = 0.004).

## 5. Conclusions

This study implements a framework for integrating vehicle trajectory data obtained from LiDAR sensors to VISSIM models and surrogate safety assessment models to predict rear-end crashes at urban signalized intersections under heterogenous traffic conditions. Vehicle trajectory data were extracted from LiDAR point clouds collected at six urban signalized intersections in Lubbock, Texas, in the USA. Each study intersection was modeled with PTV VISSIM and calibrated to replicate the observed field scenarios. Rear-end conflicts extracted from the calibrated models combined with a ten-year historical crash dataset were fitted into a Negative Binomial (NB) model to estimate the model’s parameters. The model presented offers valuable insights for implementing traffic control and safety improvement strategies at intersections. Here is how the different aspects of the model can be applied:

i. **Identifying High-Risk Intersections:** By analyzing the coefficient values for each intersection, authorities can prioritize locations with a stronger correlation between rear-end conflicts and crashes. Intersections like 50th/Quaker (high coefficient) warrant immediate attention compared to those with a lower impact (e.g., 82nd/Slide).
ii. **Targeting Safety Measures:** The model highlights rear-end conflicts as a significant factor in crashes. This knowledge allows for targeted safety measures that address these conflicts. Examples include the following:
  - **Improved Signal Timing:** Optimizing traffic light timing can reduce sudden stops and improve following distances, potentially decreasing rear-end conflicts.
  - **Advanced Warning Signs:** Flashing yellow arrows or countdown timers before red lights can warn drivers and encourage them to adjust their speed, reducing rear-end risks.
iii. **Data-Driven Decision Making:** The model can be used as a tool for the monitoring and evaluation of implemented safety measures. By analyzing changes in rear-end conflict counts after implementing interventions, authorities can assess the effectiveness of the strategies and make necessary adjustments.

The authors acknowledge some limitations of the research methodology. There was no benchmark set for the minimum throughput (volume) required for this study. Consequently, the observed volume varies significantly across all six study intersections. It is not certain how lower volumes affect the reliability of the calibration. Additionally, driver behavior is influenced by the prevailing traffic flow condition. This study did not address how changes in traffic flow condition will affect the model output. Therefore, addressing these limitations is an interesting direction of future research.

## Figures and Tables

**Table 1:** Sample of processed vehicle trajectories.

ID	Frame	Latitude	Longitude	X-Coord	Y-Coord	Link	Lane	Speed (m/s)	Acceleration (m/s^2^)
16	39	33.51976	−101.95735	46.342613	−12.572023	3	6	0.165073	−0.011634
16	40	33.51976	−101.95735	46.339467	−12.569802	3	6	0.160306	−0.004767
16	41	33.51976	−101.95735	46.349022	−12.583051	3	6	0.155536	−0.00477
16	42	33.51976	−101.95735	46.370656	−12.610098	3	6	0.144197	−0.011339
16	43	33.51976	−101.95735	46.388631	−12.630805	3	6	0.127497	−0.0167
16	44	33.51976	−101.95735	46.397277	−12.638322	3	6	0.112026	−0.015471
16	45	33.51976	−101.95735	46.399166	−12.636619	3	6	0.09962	−0.012406
16	46	33.51976	−101.95735	46.395805	−12.629402	3	6	0.083923	−0.015697
16	47	33.51976	−101.95735	46.392293	−12.624131	3	6	0.064301	−0.019622
16	48	33.51976	−101.95735	46.390476	−12.624897	3	6	0.049202	−0.015099
16	49	33.51976	−101.95735	46.389625	−12.632144	3	6	0.045827	−0.003376

**Table 2:** Definitions of the Wiedemann 99 car-following model parameters [20].

Parameter	Unit	Description
**CC0**	m	**Standstill distance:** The desired standstill distance between two vehicles.
**CC1**	s	**Gap time distribution:** Time distribution from which the gap time in seconds is drawn which a driver wants to maintain in addition to the standstill distance.
**CC2**	m	**“Following” distance oscillation:** Maximum additional distance beyond the desired safety distance accepted by a driver following another vehicle before intentionally moving closer.
**CC3**	s	**Threshold for entering “BrakeBX”:** Time in seconds before reaching the maximum safety distance to a leading slower vehicle at the beginning of the deceleration process (negative value).
**CC4**	m/s	**Negative speed difference:** Lower threshold for relative speed compared to slower leading vehicle during the following process (negative value).
**CC5**	m/s	**Positive speed difference:** Relative speed limit compared to faster leading vehicle during the following process (positive value).
**CC6**	1/(m.s)	**Distance impact on oscillation:** Impact of distance on limits of relative speed during following process.
**CC7**	m/s^2^	**Oscillation acceleration:** Acceleration oscillation during the following process.
**CC8**	m/s^2^	**Acceleration from standstill:** Acceleration when starting from standstill.
**CC9**	m/s^2^	**Acceleration at 80 km/h:** Acceleration at 80 km/h is limited by the desired and maximum acceleration functions assigned to the vehicle type.

**Table 3:** Driving behavior parameters of lane changing [20].

Element	Description
**Maximum deceleration (MaxDecelOwn and MaxDecelTrail)**	**Own (MaxDecelOwn):** Maximum deceleration for a vehicle when changing lanes, determined by its route in proximity to the emergency stop position. **Trailing vehicle (MaxDecelTrail):** Maximum deceleration for the trailing vehicle on the new lane of a changing vehicle, influenced by its route in close proximity to the emergency stop position.
**−1 m/s^2^ per distance (DecelRedDistOwn and DecelRedDistTrail)**	**Own (DecelRedDistOwn):** The distance over which the accepted deceleration for a vehicle decreases linearly by 1 m/s^2^ from the maximum deceleration when changing lanes. **Trailing vehicle (DecelRedDistTrail):** Distance over which the accepted deceleration for the trailing vehicle in the new lane of a changing vehicle is linearly reduced by 1 m/s^2^ from the maximum deceleration.
**Accepted deceleration (AccDecelOwn and AccDecelTrail)**	**Own (AccDecelOwn):** The deceleration accepted at any given time for a vehicle when changing lanes **Trailing vehicle (AccDecelTrail):** The deceleration accepted at any given time for the trailing vehicle in the new lane of a changing vehicle
**Safety distance reduction factor (lane change) (SafeDistRedFact)**	The safety distance reduction factor (lane change) (SafeDistRedFact) is taken into account for each lane change. It concerns the following parameters: The safety distance of the trailing vehicle on the new lane for determining whether a lane change will be carried out. The safety distance of the lane changer itself. The distance to the preceding, slower lane changer.
**Maximum cooperative deceleration (CoopDecel)**	Maximum cooperative deceleration (CoopDecel) specifies to what extent the trailing vehicle A is braking cooperatively, so as to allow a preceding vehicle B to change lanes into its own lane.

**Table 4:** Calibrated parameters for Macro Model.

		MACRO			
S/N	Intersection Name	CC0 (Default Value = 4.92 ft)	CC1 (Default Value = 0.9 s)	SDRF (Default Value = 0.6)	ADT (Default Value = −3.28 ft/s^2^)
1	34th Street and Indiana Avenue	6.7	0.8	0.3	−6.56
2	82nd Street and Milwaukee Avenue	6.7	0.9	0.4	−6.56
3	82nd Street and Slide Road	2.6	0.9	0.1	−3.28
4	50th Street and Avenue Q	3.7	0.9	0.1	−3.28
5	50th Street and Quaker Avenue	4.5	0.8	0.2	−3.28
6	4th Street and Frankford Avenue	4.5	0.8	0.6	−6.56

**Table 5:** Calibrated parameters for Micro Model.

		MICRO			
S/N	Intersection Name	CC0 (Default Value = 4.92 ft)	CC1 (Default Value = 0.9 s)	SDRF (Default Value = 0.6)	ADT (Default Value = −3.28 ft/s^2^)
1	34th Street and Indiana Avenue	5.5	0.7	0.5	−6.56
2	82nd Street and Milwaukee Avenue	3.1	0.9	0.4	−1.64
3	82nd Street and Slide Road	3.5	1	0.2	−1.64
4	50th Street and Avenue Q	6.3	1	0.3	−3.28
5	50th Street and Quaker Avenue	6.1	0.8	0.2	−1.64
6	4th Street and Frankford Avenue	1.9	0.7	0.3	−6.56

**Table 6:** Observed versus simulated traffic volumes for the study intersections.

S/N	Intersection Name	Observed Volume from LiDAR (veh/h)	Simulated Volume (veh/h)					
			Default Model	Accuracy %	Macro Model	Accuracy %	Micro Model	Accuracy %
1	34th Street and Indiana Avenue	5727	5689	99.3	5717	99.8	5721	99.9
2	82nd Street and Milwaukee Avenue	5967	5804	97.3	5962	99.9	5962	99.9
3	82nd Street and Slide Road	5300	5286	99.7	5291	99.8	5288	99.8
4	50th Street and Avenue Q	4459	4309	96.6	4449	99.8	4455	99.9
5	50th Street and Quaker Avenue	6080	5776	95.0	6055	99.6	6050	99.5
6	4th Street and Frankford Avenue	3500	3206	91.6	3475	99.3	3489	99.7

**Table 7:** Observed mean speeds versus simulated mean speeds.

S/N	Intersection Name	Observed Mean Speed from LiDAR (mi/h)	Simulated Speed (mi/h)					
			Default Model	Accuracy %	Macro Model	Accuracy %	Micro Model	Accuracy %
1	34th Street and Indiana Avenue	20.88	14.45	69.2	17.54	84.00	19.72	94.4
2	82nd Street and Milwaukee Avenue	22.81	16.95	74.3	19.63	86.1	22.05	96.7
3	82nd Street and Slide Road	21.69	15.78	72.8	17.04	78.6	21.13	97.4
4	50th Street and Avenue Q	22.95	18.46	80.4	18.80	81.9	20.15	87.8
5	50th Street and Quaker Avenue	22.53	19.94	88.5	20.80	92.3	21.38	94.9
6	4th Street and Frankford Avenue	22.80	17.85	78.3	18.22	79.9	20.83	91.4

**Table 8:** Extracted rear-end conflicts at the study intersections.

S/N	Intersection Name	Rear-End Conflict		
		Default Model	Macro Model	Micro Model
1	34th Street and Indiana Avenue	702	811	993
2	82nd Street and Milwaukee Avenue	615	628	1194
3	82nd Street and Slide Road	539	834	1052
4	50th Street and Avenue Q	512	618	932
5	50th Street and Quaker Avenue	417	417	623
6	4th Street and Frankford Avenue	474	485	604

**Table 9:** Negative Binomial Model results for the study intersections.

Model Output	34th/Indiana	82nd/Milwaukee	82nd/Slide	50th/Ave Q	50th/Quaker	4th/Frankford
Constant (Intercept)	−2.2513	−2.2524	−1.0561	−1.7797	−0.8329	−4.7538
Rear-End Conflict Count	0.2214	0.0423	0.0165	0.6979	1.5677	0.4993
Pseudo R-squared (Cox and Snell)	0.6515	0.5496	0.2142	0.3768	0.354	0.5834
*p*-value	0.000	0.000	0.050	0.000	0.018	0.004
Akaike Information Criterion (AIC)	28.4242	30.1829	41.8509	34.5000	47.3026	16.669
Log-Likelihood	−12.212	−13.091	−18.925	−15.250	−21.651	−6.3345
Deviance	11.091	13.253	25.302	18.366	29.001	1.9527

## Data Availability

Data will be made available on request.
